# Intellectual Disability among Dutch Homeless People: Prevalence and Related Psychosocial Problems

**DOI:** 10.1371/journal.pone.0086112

**Published:** 2014-01-21

**Authors:** Barbara Van Straaten, Carola T. M. Schrijvers, Jorien Van der Laan, Sandra N. Boersma, Gerda Rodenburg, Judith R. L. M. Wolf, Dike Van de Mheen

**Affiliations:** 1 Erasmus Medical Centre, Rotterdam, the Netherlands; 2 IVO Addiction Research Institute, Rotterdam, the Netherlands; 3 Radboud University medical center, Department of Primary and Community Care, Netherlands Center for Social Care Research, Nijmegen, the Netherlands; Chiba University Center for Forensic Mental Health, Japan

## Abstract

**Background:**

There is a higher prevalence of intellectual disability (ID) among homeless people than in the general population. However, little is known about the additional psychosocial problems faced by homeless people with ID. We describe the prevalence of ID in a cohort of homeless people in the Netherlands, and report relationships between ID and psychosocial problems in terms of psychological distress, substance (mis)use and dependence, as well as demographic characteristics in this cohort.

**Methods:**

This cross-sectional study is part of a cohort study among homeless people in the four major cities of the Netherlands. Data were derived from 387 homeless people who were interviewed and screened for ID six months after the baseline measurement. Multivariate logistic regression analyses and χ^2^ tests were performed to analyze relationships between ID, psychosocial problems and demographic characteristics.

**Findings:**

Of all cohort members, 29.5% had a suspected ID. Participants with a suspected ID had a higher mean age, were more likely to be male and to fall in the lowest category of education than participants without a suspected ID. Having a suspected ID was related to general psychological distress (OR  = 1.56, *p*<0.05), somatization (OR  = 1.84, *p*<0.01), depression (OR  = 1.58, *p*<0.05) and substance dependence (OR  = 1.88, *p*<0.05). No relationships were found between a suspected ID and anxiety, regular substance use, substance misuse and primary substance of use.

**Conclusion:**

The prevalence of ID among Dutch homeless people is higher than in the general population, and is related to more psychosocial problems than among homeless people without ID. Homeless people with a suspected ID appear to be a vulnerable subgroup within the homeless population. This endorses the importance of the extra attention required for this subgroup.

## Introduction

Apart from the lack of housing, being homeless is related to a number of additional problems. Studies have shown higher rates of mental health problems and substance use problems among homeless people as compared with the general population [1]. A more recent topic of interest in the field of research on homelessness is the prevalence of (mild) intellectual disability (ID) (IQ <70). A systematic review on cognitive dysfunction in homeless adults shows that 30–40% of homeless adults have a cognitive impairment [2]. In another study, 12% of 50 homeless people met the criteria for ID [3]. Compared to the prevalence of ID in the general Dutch population, which is about 0.7% [4], the prevalence reported among homeless populations is (very) high. However, sample sizes in previous studies are relatively small and most included only homeless people living in a specific facility, which can limit the generalizability of these prevalence estimates to other homeless populations. Also, most of the earlier studies were conducted in the USA and the UK, where the occurrence of homelessness and social welfare systems differ substantially from most (other) European countries [5].

Apart from the prevalence of ID in homeless populations, it is highly relevant to study related psychosocial problems among homeless people with ID. More insight in the situation of homeless people with ID may contribute to the development of services that fit the needs of this specific, and presumably fairly large, subgroup. A study on a general (non-homeless) population with ID reported a lower prevalence of alcohol and drug use but a potentially elevated risk of experiencing a substance use disorder among people with ID [6]. Also, it was found that (non-homeless) people with ID have a higher rate of mental health problems than the general population [7]–[9]. In a large population-based study, 31.7% of people with an ID also had a psychiatric disorder [10].

To our knowledge, only one study has described the characteristics and problems of homeless people with ID and compared them with homeless people without ID [11]. In that study the proportion of women was higher in the group of homeless people with ID, but no differences were found between the two groups with regard to mental health problems and substance abuse. However, these results seem in contrast to earlier reports of more substance-use disorders and mental health problems in general populations with ID as compared with those without ID. Thus, until now, it remains unclear whether homeless people with ID also have additional problems.

The first aim of this study is to examine the prevalence of ID among Dutch homeless people. We hypothesize that the prevalence of ID in this group is higher than the 0.7% found in the general Dutch population [4]. The second aim is to explore relationships between ID and psychosocial problems frequently seen in homeless populations: psychological distress and substance (mis)use dependence. This study is part of the ‘Cohort study amongst homeless people in Amsterdam, The Hague, Rotterdam and Utrecht’, which follows homeless people for a period of 2.5 years from the moment they reported themselves at a central access point for social relief in 2011 in one of the four major cities in the Netherlands (Amsterdam, The Hague, Rotterdam and Utrecht).

## Methods

### Ethics statement

The study complies with the criteria for studies which have to be consulted by an accredited Medical Research Ethics Committee (aMREC). Upon consultation, the Medical Review Ethics Committee region Arnhem-Nijmegen concluded that ethical approval was not necessary (Registration number 2010/321). The study was conducted according to the principles expressed in the Code of Conduct for health research with data (http://federa.org). All participants gave written informed consent.

### Design and participants

This cross-sectional study is part of a larger observational longitudinal multi-site cohort study following homeless people for a period of 2.5 years, starting from the moment they reported themselves at a central access point for social relief in 2011 in one of the four major cities in the Netherlands (Amsterdam, The Hague, Rotterdam and Utrecht) and were accepted for an individual programme plan. It is obligatory for every homeless person to report at a central access point for social relief in order to get access to social relief facilities, such as a night shelter. The aim of the cohort study is to determine predictors of an improved quality of life and stable housing among homeless people, and to explore their experiences with a person-oriented approach. This person-oriented approach is part of the Strategy Plan for Social Relief, a Dutch policy aimed at preventing and reducing homelessness, and improving the situation of homeless people, by offering them an individual programme plan. We included the homeless people from the four major cities in the Netherlands because they all work with the same policy regarding homeless people, namely the person-oriented approach, and in order to get a large enough sample size to obtain our research aim.

All 513 study participants satisfied the criteria set by the four major cities in the Netherlands for starting an individual programme plan. These include: being at least 18 years of age, having legal residence in the Netherlands, residing in the region of application for at least two years during the last three years, having abandoned the home situation, and being unable to hold one's own in society. Consequently, other subgroups (such as illegal homeless people) were excluded from this study. The participants, consisting of homeless adults (aged ≥23 years) and homeless youth (aged 18–22 years), were divided over the four cities in accordance with the inflow of homeless people at the central access points for social relief.

We compared the total group of homeless adults and youth who reported themselves at a central access point for social relief in one of the four major cities in the Netherlands in 2011 with the study participants. Adult participants (aged ≥23 years; *n* = 410) were representative in terms of age and gender. Youth participants (aged 18–22 years; *n* = 103) were representative in terms of age, but in this subgroup males were overrepresented (60.2% younger males in the cohort vs. 49.2% younger males in the total group). This constitutes the subgroup of homeless people in the four major cities in the Netherlands who are included in this study.

The cohort study has a follow-up period of 2.5 years. After the baseline interview (T0), participants were interviewed an additional three; after 6 months (T1), after 18 months (T2), and after 36 months (T3). The cross-sectional data in this study are derived from the second interview (T1), which took place between July 2011 and June 2012.

### Procedure and study sample at first measurement

At the start of the study in January 2011, potential participants were approached either at a central access point for social relief (one in each city) by an employee of the access point, or at a temporary accommodation where they stayed shortly after entering the social relief system by the researchers or interviewers. Potential participants were informed about the study by means of leaflets, posters and face-to-face information provision. When a potential participant expressed interest in taking part in the study, the researchers contacted that person to explain the aim of the study, the procedure of the interview and including informed consent. When the informed participant agreed to participate in the study on the term explained to them, an interview appointment was scheduled.

A trained interviewer met the participant at the participant's location of choice (most often a shelter facility, public library, or the researcher's office). All participants gave written informed consent. Participants were interviewed face-to-face by using a structured questionnaire (mean duration of 1.5 h) and received €15 (± $19) for their participation. The interviews were held in Dutch, English, Spanish or Arabic.

We anticipated on problems that may occur when using questionnaires designed for the general population among people with ID (e.g. acquiescence, not understanding the question, getting tired during the interview). Participants were told at the start of the interview that they could take a break during the interview whenever they wanted to. They were allowed for missing answers in case they did not know what to answer or did not want to answer (a ‘don't know’ and a ‘no answer’ option was present and were regarded as missing, as is recommended for the use of questionnaires on people with intellectual disabilities [12]). We presented the questionnaires orally to take into account participants who may have trouble with reading. Also, regarding the questionnaires with a multiple-choice format, we presented cards with the answering categories listed to the participant (for example ‘not at all’; ‘a little bit’, etc.) and we repeated the categories verbally when needed. Also, all interviewers were given an interviewer manual with more easy to understand synonyms for potentially difficult words used in the questionnaire. When in doubt whether a participant did or did not understand the question, interviewers repeated the question or used the synonyms, as suggested in the manual.

### Procedure and study sample at second measurement

Participants were contacted for the second measurement 6 months after the first measurement by telephone, e-mail, letter, their social contacts, their caregiver/institution, or private messages via social media. Prior to the baseline interview, they had provided this contact information and had agreed that it could be used to contact them for the second interview. Participants were interviewed in the same way as during the first measurement: face-to-face, with a structured questionnaire (mean duration of 1.5 h), and with the same support options (optional break during the interview, cards with answering categories, etc). The participants received €20 (± $26) for their participation.

Of the initial cohort of 513 participants, 396 (77.2%) were interviewed for the second measurement. We compared them with non-participants (*n* = 117; 22.8%) of the second measurement on demographic variables, substance use and psychological distress as reported at the first measurement. Compared with participants, non-participants were more regular users of cannabis (35.0% vs. 25.0%), were on average younger (33.3 years vs. 37.2 years) and more often had primary education only (42.2% vs. 31.6%).

For the purpose of the current study, we excluded participants who did not complete the screener for intellectual disability (*n* = 9). Five of them were not screened for ID because of a language barrier and four participants refused to be screened for ID. Therefore, the situation of 387 adults and youth is described at the second measurement.

### Measurements

#### Demographic characteristics

Demographic characteristics including gender, age, ethnicity and educational level were assessed. Age was calculated by subtracting the date of birth from the date on which the second measurement took place. Ethnicity was categorized into ‘native Dutch’ when the participant and both parents were born in the Netherlands, ‘first-generation immigrants’ when participants were foreign born, and ‘second-generation immigrants’ when participants were born in the Netherlands but one or both of their parents were foreign born.

Education was categorized as ‘lowest’ when the participant completed primary education at the most, as ‘low’ when the participant completed pre-vocational education, lower technical education, assistant training or basic labour-oriented education, as ‘intermediate’ when the participant completed secondary vocational education, senior general secondary education or pre-university education, and categorized as ‘high’ when the participant completed higher professional education or university education.

#### Intellectual disability (ID)

To measure a suspected ID, the Hayes Ability Screening Index (HASI) [13] was used. The HASI is a brief, individually administered screening index of intellectual abilities. It was initially developed to indicate the possible presence of ID among people in contact with the criminal justice system and was designed to be culture-fair. Because it is not a full-scale diagnostic instrument in itself, it only gives an indication of whether a person has an ID (IQ <70) and whether full-scale diagnostic assessment is recommended.

The index consists of four subtests: background items, backwards spelling, a puzzle and clock drawing, and can be administered in 5–10 min. The HASI shows a significant correlation with other psychometric tests measuring cognitive ability (0.627 for the Kaufman Brief Intelligence Test (K-BIT), 0.497 for the Vineland Adaptive Behavior Scales (VABS) [13]. A HASI cut-off score of 85 was found to be the optimum for discriminating between participants with and without a suspected ID, with a sensitivity of 82.4 and specificity of 71.6 [13]. This is the cut-off score we used in this study to distinguish the group ‘suspected ID’ (HASI score below 85, corresponding to an IQ <70) and ‘no suspected ID’ (HASI score of 85 or more, corresponding to an IQ ≥70). We used the Dutch version of the HASI, which was translated and provided by the developers of the HASI.

#### Psychological distress

The Brief Symptom Inventory 18 (BSI-18) was used to measure psychological distress [14]. The BSI-18 is a short form consisting of 18 items taken from the Symptom Checklist-90-R (SCL-90-R) [15], which correlates highly with the SCL-90-R. The BSI-18 assesses three symptom scales; Somatization, Depression and Anxiety, and includes a total score as an indication of general psychological distress. The BSI is a frequently used measure to evaluate psychological distress in studies among homeless populations [16]–[20]. The Dutch translation was used, with (provisional) norm scores for the Dutch population [21]. We compared the scores of the participants with the norm scores described in the manual for the Dutch community sample, with separate norm scores for men and women, and for different age categories (18–29 years and 30+ years) [21]. Participants were categorized into two groups: participants with a normal score and participants with an elevated score on the BSI-18. Because norms for t-scores are not available for the Dutch BSI-18 [21], participants were categorized as having an elevated score if they scored in the upper 40^th^ percentile on a subscale or on the total score compared with a Dutch community sample. The use of this cut-off point allowed us to maintain statistical power, because by using this cut-off score the two groups were approximately equally divided.

In accordance with the manual instructions, we excluded participants who did not answer all questions that compose a certain subscale score (maximum *n* = 2 per subscale) or the total score (*n* = 3).

#### Substance use, misuse and dependence

Substance (mis)use and dependence were assessed using the Measurements in the Addictions for Triage and Evaluation (MATE) [22]. The MATE is a measurement tool for assessing characteristics of people with drug and/or alcohol problems for triage and evaluation in treatment. The MATE has satisfactory inter-rater reliability (range 0.75–0.92), but less satisfactory test-retest reliability (0.34–0.73) [23].

For the present study only one of the 10 original modules of the tool was used, which is module four: ‘Substance dependence and abuse’. This module consists of 11 questions from the Composite International Diagnostic Interview (CIDI) [24]. Two examples of those questions are: ‘In the past 12 months, did you find you began to need much more [substance] to get the same effect or that the same amount of [substance] had less effect than it once had?’ and ‘In the past 12 months, has your use of [substance] led to problems with the police?’.

After consultation with the developers of the MATE, we added a screening question to the module to select only those participants who regularly (at least once a week) used a substance in the past 12 months before the interview took place. For statistical purposes we made three categories of primary substance of use: mainly alcohol, mainly cannabis and mainly other substances. The last category consisted of a collection of hard drugs (cocaine, methadone, heroin, XTC, amphetamine), as the use of those substances was too rare for meaningful separate analyses.

The score for dependence was calculated by the sum of positive answers on the first seven items from module four, the score for abuse is calculated by the sum of positive answers on the last four items of module four. In accordance with the DSM-IV [25], a participant was classified as ‘substance dependent’ when he/she had three or more positive answers on the seven dependence items. A participant was classified as ‘substance misuser’ when he/she had one or more positive answers on the four misuse items.

### Statistical analysis

Descriptive analyses were performed to describe the prevalence of ID, demographic characteristics, psychological distress, regular substance use, substance misuse, dependency and primary substance of use for the group with and without a suspected ID. Relationships between ID and demographic characteristics were analyzed using χ^2^ tests for categorical data (gender, education, ethnicity) and a t-test for the continuous variable (age). Relationships between ID and psychological distress were tested using logistic regression. Relationships between ID and regular substance use, substance misuse, substance dependence and primary substance of use were tested using multivariate logistic regression. In all logistic regression analyses, we controlled for age and gender, except for ‘psychological distress’ because we used age- and gender-specific percentile scores (see Measurements), which makes additionally adjusting for age and gender superfluous. The results are reported as odds ratios (OR) with 95% confidence intervals (CI) and *p*-values. The reported *p*-values are two-sided and level of significance was set at *p*<0.05. All statistical analyses were conducted with the statistical software package IBM SPSS statistics version 19.

## Results

### Prevalence of a suspected ID

Of the 387 participants, 114 (29.5%) had a suspected ID.

### Characteristics of participants with and without a suspected ID

Table 1 presents characteristics of participants with and without a suspected ID. The mean age of participants with a suspected ID was significantly higher than that of those without a suspected ID, and significantly more participants with a suspected ID were male. The overall χ^2^ test indicated a significant relation between a suspected ID and education level. Participants with a suspected ID were more likely to fall in the lowest category of education, and less likely to fall in the low or intermediate category. For ethnicity, no significant difference between participants with and without a suspected ID was found.

**Table 1:** Demographic characteristics of participants with and without a suspected ID.

	Suspected ID	No suspected ID	*p-value*
**Mean age in years (sd)** (*n* = 387)	39.9 (13.0)	36.6 (13.2)	t (385) = −2.294; ***p*** ** = 0.022**
**Gender % male** (*n* = 387)	84.2	71.8	?^2^ (1) = 6.693; ***p*** ** = 0.010**
**Education %** (*n* = 384)			?^2^ (3) = 21.414; ***p*** **<0.001** a
Lowest	44.6	25.4	
Low	37.5	50.0	
Intermediate	6.3	17.6	
High	11.6	7.0	
**Ethnicity %** (*n* = 379)			?^2^ (2) = 3.037; *p* = 0.219
Native Dutch	34.8	39.7	
First-generation immigrant	47.3	37.8	
Second-generation immigrant	17.9	22.5	

*p*-values in bold indicate a significant difference (*p*<0.05); ID  =  intellectual disability; sd  =  standard deviation

a Post-hoc *χ* ^2^: Lowest; ID > no ID; *χ* ^2^ (1)  =  13.782, *p*<0.001, OR  =  2.27, CIs [1.495–3.766] Low; ID < no ID; *χ* ^2^ (1)  =  4.985, *p*<0.05, OR  =  0.60, CI [0.382, 0.941] Intermediate; ID < no ID; *χ* ^2^ (1) 8.397, *p*<0.01, OR  =  0.31, CI [0.136–0.711].

### Relationships between a suspected ID and psychological distress

Descriptive analyses provided the percentage of elevated scores on psychological distress of participants with and without a suspected ID; for both groups, the percentage of elevated scores was highest for somatization (60.2% and 45.1%, respectively). On all subscales and general psychological distress, participants with a suspected ID had a higher percentage of elevated scores (Table 2). Participants with a suspected ID had higher odds of having an elevated score on somatization (OR  = 1.84, *p* = 0.007), depression (OR  =  1.58, *p* = 0.044), and general psychological distress (OR  = 1.56, *p* = 0.049) than participants without a suspected ID. No significant relation was found between a suspected ID and elevated anxiety scores.

**Table 2:** Relationships between suspected ID and elevated psychological distress scores in homeless people.

	Suspected ID	No suspected ID	OR	95% CI	*p-value*
**Somatization** (*n* = 386) % elevated somatization score	60.2	45.1	1.84	1.180–2.878	**0.007**
**Depression** (*n* = 385) % elevated depression score	49.1	38.0	1.58	1.013–2.449	**0.044**
**Anxiety** (*n* = 386) % elevated anxiety score	51.8	42.6	1.44	0.930–2.238	0.101
**Total BSI-18 score** (*n* = 384) % elevated general distress score	57.5	46.5	1.56	1.001–2.427	**0.049**

Note: for each comparison, the no suspected intellectual disability group is the reference group.

*p*-values in bold indicate a significant relationship (*p*<0.05) ID  =  intellectual disability.

### Relationships between a suspected ID and substance (mis)use and dependence

Table 3 shows that participants with a suspected ID had almost two times greater odds of being classified as substance dependent than participants without a suspected ID (OR  = 1.88, *p* = 0.021). Table 4 shows that regular substance users mainly used alcohol or cannabis. No significant relationships were found between a suspected ID and regular substance use in the past 12 months, substance misuse (Table 3) and primary substance of use (Table 4).

**Table 3:** Relationships between suspected ID and regular substance use, substance misuse and substance dependence in homeless people*.

	Suspected ID	No suspected ID	OR	95% CI	*p-value*
**Regular substance use in the past 12 months (%)** (*n* = 387)	51.8	44.7	1.29	0.812–2.046	0.281
**Substance misuse** (%) (*n* = 386)	31.6	25.7	1.30	0.782–2.146	0.314
**Substance dependence** (%) (*n* = 386)	28.9	18.4	1.88	1.102–3.206	**0.021**

*Multivariate logistic regression analysis adjusted for age and gender

Note: for each comparison, the no suspected ID group is the reference group.

*p*-values in bold indicate a significant relationship (*p*<0.05) ID  =  intellectual disability.

**Table 4:** Relationships between suspected intellectual disability (ID) and primary substance of use in homeless people who regularly use substances (*n* = 180)*.

	Suspected ID	No suspected ID	OR	95% CI	*p-value*
**Mainly alcohol use (%)**	55.9	47.9	1.25	0.637–2.467	0.512
**Mainly cannabis use (%)**	30.5	46.3	0.53	0.251–1.101	0.088
**Mainly other substances (%) (1)**	13.6	5.8	2.46	0.836–7.247	0.102

*Multivariate logistic regression analysis adjusted for age and gender.

(1) Other substances: cocaine (*n* = 12), methadone (*n* = 2), heroin (*n* = 1), XTC (*n* = 1), amphetamine (*n* = 1).

ID  =  intellectual disability.

Note: for each comparison, the no suspected ID group is the reference group.

## Discussion

As hypothesized, this study on Dutch homeless people who reported themselves at a central access point for social relief indicates that the prevalence of ID among homeless people is higher (29.5%) than that of ID in the general Dutch population (0.7%) [4]; this is in line with data from similar prevalence studies on ID among homeless populations [2], [3]. Regarding psychosocial problems, relationships were found between ID and elevated levels of somatization, depression and general psychological distress, but not between ID and elevated levels of anxiety. In addition, homeless people with a suspected ID are more likely to be substance dependent than homeless people without a suspected ID, but in general do not report more substance use. These findings are also consistent with other studies among non-homeless populations [6], [7].

Several biological, psychological, social and developmental factors may account for the higher prevalence rates of psychological distress seen in people with ID in the general population, as well as in this homeless population [7]. International prevalence studies revealed that people with mental illness have significantly higher rates of substance use disorders than the general population [26]. This implies that the higher percentage of homeless people with ID classified as substance dependent as compared to those without ID, might be explained by the higher percentage of homeless people with ID with elevated scores on psychological distress. The elevated scores on psychological distress may (in part) be caused by the more limited coping strategies related to people with ID [27]. However, as with all cross-sectional studies, cause and effect could not be distinguished.

Anxiety was the only psychological factor for which homeless people with a suspected ID did not differ from homeless people without a suspected ID. It was earlier proposed that adults with ID may be less sensitive to anxiety; more specifically, that panic disorder may be less prevalent in adults with ID due to lack of the cognitions required to develop panic attacks [28], which may explain this result.

Besides the relationships found between ID and psychosocial problems, we also found relationships with gender and age. An explanation for the finding that participants with a suspected ID had a higher mean age, may be that the older participants have more prolonged exposure to stress than the younger participants, which may have negatively influenced their cognitive abilities [29]. Also, the prolonged use of substances among older participants compared with younger participants may partly explain the higher mean age of participants with ID. The effect for gender, namely that the percentage of men is higher among those with an ID than among those without an ID, might be attributed to differences in substance use related to gender, i.e. males were more often regular substance users than females. In the present population alcohol was the most frequently used substance and heavy use of alcohol is related to poorer performance on cognitive tasks [30]. An earlier study comparing homeless people with and without ID reported more women in the group of homeless people with ID as compared to those without; however, this latter finding is likely explained by methodological issues [11]. In the general adult population with ID, gender differences are not evident [31].

The present study has a number of strengths. First, it is one of the few to investigate relationships between ID and psychosocial problems among homeless people. In addition, in relation to an investigation of ID among homeless people, the current sample size is one of the largest to date. Thirdly, we used the HASI [12], which is a measure originally developed for a vulnerable group (i.e. people in contact with the criminal justice system). The validity of this measure to screen ID was confirmed in the present study: for example, belonging to the lowest category of education was strongly and significantly related to a suspected ID. Within the group with a suspected ID, 44.6% had the lowest level of education whereas in those without a suspected ID 25.4% had the lowest level of education. Furthermore, the ID screener was designed to be culture-fair; this factor is important for the present study as 61.7% of our participants were immigrants. Because we found no relationship between a suspected ID and being an immigrant, this probably confirms the cultural-fairness of the screener.

However, because the ID screener was designed to be over-inclusive [12], a relatively large number of false-positives might have occurred. A recent validation study on the Dutch version of the HASI suggested to lower the cut-off score from 85 to 81 to prevent potential unnecessary referrals to care institutions [32]. However, for screening in a research setting this drawback is less important. Also, the inclusion of people with borderline ID (IQ 70–85) as having a suspected IQ (instead of only those with an IQ <70) as a result of over-inclusiveness is acceptable in the present study, as those people also need to be taken into account within a homeless population.

Another point is that participants might have screened positive on ID as a result of their substance use. It was earlier suggested that (heavy) substance use is a cause of cognitive impairment [33] which may imply that ID as a developmental disorder originated before age 18 years [34] could not be confirmed in some of the present participants. On the other hand, the result of the ID screener does represent the level at which they are currently functioning, which may have implications for their situation and care needs. In addition, a part of the ID screener also consists of background questions during the school-age period, e.g. attendance at a special school. These aspects are not likely to be caused by substance use as they reflect the situation in their school-age period. However, full-scale assessment of IQ is a recommended next step in the practice of care after a positive screening result on ID, to provide efficient and tailored care. Also for future research it would be very informative to administer a full IQ test to gain a more in-depth insight in the relationship between ID and psychosocial problems and to evaluate possible dose-response relationships.

With regard to psychological distress, we chose a cut-off of the upper 40^th^ percentile on the BSI-18 to distinguish between participants with a normal score and participants with an elevated score. Even though our categorization of elevated psychological distress is not clinically relevant, this categorization of people who experience elevated psychological distress can be helpful for professionals working with homeless people as an indication that a person may have psychological problems and may need to be further examined. An interesting topic for further research concerning the relationship between psychological distress and ID, would be to investigate whether the pattern of this relationship is similar in the homeless population as compared to the non-homeless population.

Another methodological concern is related to the subgroup of the total population of homeless people in the Netherlands that was studied, i.e. only those who reported themselves at a central access point for social relief in 2011 in one of the four major cities in the Netherlands and were accepted for an individual programme plan. As stated before, it is obligatory for every homeless person to report oneself at a central access point for social relief in order to gain access to social relief facilities (such as a night shelter). Therefore, a substantial part of the homeless population is covered when using this selection criterion. Subgroups of homeless people not included in this study were illegal homeless people and homeless people who do not make use of social relief facilities. Therefore, our findings may not be representative of these latter subgroups of the Dutch homeless population. Another issue is the selective non-response of participants with a low level of education and cannabis use. This could have resulted in an underestimation of the prevalence of ID and of substance (mis)use and dependence. The amount of this underestimation can however not be calculated. In addition, we relied on self-reports to select participants who regularly used substances, which may have led to an underestimation of consumption of substances. Nevertheless, in the general population self-report measures have shown reasonable levels of reliability and validity when measuring alcohol consumption [35] and cannabis consumption [36]. Also, it has been suggested that people with ID are able to provide valid data on substance use [37]. Although no such studies exist for homeless populations, this allowed us to conclude that self-report is a valid and reliable measure for our purposes. The validity and reliability of using questionnaires designed for the general population among people with ID might be an issue. However, adequate item reliability and discriminative validity of the BSI-18 could be assumed based on research validating the use of the SCL-90-R among people with ID [38]. Also, other problems may occur using questionnaires designed for the general population among people with ID (e.g. acquiescence, not understanding the question, getting tired during the interview). We anticipated on these problems in several ways as is described in the Methods section.

## Conclusion

To our knowledge this is the first study to explore relationships between ID and psychosocial problems among homeless people in the Netherlands. The study shows that ID is indeed a relevant problem among these homeless. It also indicates that ID screening of homeless people may be an effective method to identify those who are particularly vulnerable in terms of psychosocial problems within a homeless population. In this subgroup, the additional mental health and substance use problems may have implications for care programmes and homeless services, and endorses the importance of the extra attention required for this subgroup. This subgroup may benefit from customised care programmes and specialised housing facilities designed for homeless people with ID. The relatively large number of homeless people with ID emphasises that expertise in the field of ID among professionals working in homeless services is required. Further research on the care needs and service use of homeless people with ID is needed to improve the living situation of one of the most vulnerable groups in society.
